# Compelling Long-Term Results for Liver Resection in Early Cholangiocarcinoma

**DOI:** 10.3390/jcm10132959

**Published:** 2021-06-30

**Authors:** Jan Bednarsch, Zoltan Czigany, Lara R. Heij, Dong Liu, Marcel den Dulk, Georg Wiltberger, Philipp Bruners, Tom Florian Ulmer, Ulf Peter Neumann, Sven Arke Lang

**Affiliations:** 1 Department of Surgery and Transplantation, University Hospital RWTH Aachen, 52074 Aachen, Germany; jbednarsch@ukaachen.de (J.B.); zczigany@ukaachen.de (Z.C.); lheij@ukaachen.de (L.R.H.); dliu@ukaachen.de (D.L.); marcel.den.dulk@mumc.nl (M.d.D.); gwiltberger@ukaachen.de (G.W.); fulmer@ukaachen.de (T.F.U.); uneumann@ukaachen.de (U.P.N.); 2 Institute of Pathology, University Hospital RWTH Aachen, 52074 Aachen, Germany; 3 Department of Surgery, Maastricht University Medical Center (MUMC), 6229 Maastricht, The Netherlands; 4 Department of Diagnostic and Interventional Radiology, University Hospital RWTH Aachen, 52074 Aachen, Germany; pbruners@ukaachen.de

**Keywords:** intrahepatic cholangiocarcinoma, surgery, liver transplantation

## Abstract

Surgery for intrahepatic cholangiocarcinoma (iCCA) is associated with a high rate of recurrence even after complete resection. To achieve acceptable results, preoperative patient selection is crucial. Hence, we aimed to identify preoperative characteristics with prognostic value focusing on certain radiological features. Patients who underwent hepatectomy for iCCA between 2010 and 2020 at University Hospital, RWTH Aachen were included. Kaplan–Meier and Cox regressions were applied for survival analysis and associations of overall survival (OS) and recurrence-free survival (RFS) with clinical/radiological characteristics, respectively. Based on radiological features patients were stratified into three groups: single nodule ≤ 3 cm, single nodule > 3 cm, and ≥2 nodules. Analysis of 139 patients revealed a mean OS of 142 months for those with a single nodule ≤3 cm, median OS of 28 months with a single nodule >3 cm, and 19 months with ≥2 nodules, respectively. Multivariable analyses based on preoperative characteristics showed the radiological stratification to be independently associated with OS (HR (hazard ratio) = 4.25 (1 nodule, >3 cm), HR = 5.97 (≥2 nodules), *p* = 0.011), RFS (HR = 4.18 (1 nodule, >3 cm), and HR = 11.07 (≥2 nodules), *p* = 0.001). In conclusion, patients with single iCCA ≤3 cm show compelling OS and RFS. Basic radiological features (e.g., nodule size, number) are prognostic for patients undergoing surgery and useful in preoperative patient selection.

## 1. Introduction

Cholangiocellular carcinoma (CCA) is subclassified into intrahepatic CCA (iCCA), perihilar CCA (pCCA), and distal CCA (dCCA) with pCCA being the most frequent followed by dCCA and iCCA [1]. iCCA comprises about 15% of primary liver tumors and, therefore, is regarded to be the second most common primary liver cancer [2]. Over the last decades, an increasing incidence of iCCA has been reported whereas incidence rates for pCCA and dCCA are slightly declining [3,4]. While chronic inflammation and bile stasis are common risk factors for all subtypes of CCA, other risk factors such cirrhosis, hepatitis B/C, and alcohol consumption seem to be more important in the development of iCCA [5]. The latter is of particular importance, since these risk factors can significantly affect liver function which might also limit the feasibility of therapies proposed for iCCA.

CCAs of either localization are often diagnosed in advanced stages requiring complex medical, interventional, or surgical treatment. In the absence of distant metastasis, radical surgery with lymphadenectomy is the gold standard for each subtype of CCA offering superior oncological outcome over medical or interventional treatment [6,7]. Surgery for iCCA and pCCA often requires extended liver resections with the concomitant resection and reconstruction of major vascular and biliary structures to achieve local tumor clearing (R0 situation) [8]. One of the major challenges in this context is the risk of posthepatectomy liver failure (PHLF), which dramatically increases in the presence of preexisting liver damage such as fibrosis, cirrhosis, or nonalcoholic steatohepatitis (NASH) [7]. In fact, surgery for i/pCCA can result in significantly higher perioperative morbidity and mortality compared to surgery for other gastrointestinal malignancies, although efforts have been made to identify patients at risk for PHLF [7,8,9,10,11]. In case of major liver resections, preoperative volumetry is routinely used to assure that enough future liver remnant (FLR) remains after resection to prevent PHLF [12]. In addition, liver function can be estimated using dynamic liver function tests such as indocyanine green (ICG) clearance or LiMAx test and imaging-based methods (e.g., ^99m^Tc-Mebrofenin and MRI with Gd-EOB-DTPA) [13]. More recently, parameter such as sarcopenia, albumin-bilirubin (ALBI) score, and liver/spleen stiffness have been used to assess the risk of PHLF [14,15,16]. Notably, these parameters can also be used to evaluate long-term prognosis after resection of primary liver tumors including iCCA [17,18,19]. Nonetheless, some patients might still be precluded from potentially curative surgery due to structural liver damage and, therefore, the need for novel treatment options is urgent. 

The combination of impaired parenchymal quality/function and the need for (often extended) liver resection soon brought up the idea of orthotopic liver transplantation (OLT) as therapy for iCCA. However, results from early case series were disappointing with 5-year OS between 0 and 30% and subsequently OLT was abandoned for iCCA for decades [20,21,22]. Nonetheless, a number of studies suggest a role for OLT in selected cases with early pCCA which are not eligible for liver resection due to technical irresectability or underlying liver cirrhosis, particularly after neoadjuvant treatment [20,23,24]. Regarding iCCA, recently published data from incidentally detected tumors have changed the few on OLT as a treatment option. In fact, retrospective analyses from Spain and Japan showed 5-year OS between 45 and 55% in these patients [25,26]. Furthermore, a retrospective multicenter follow-up study by Sapisochin et al. confirmed these initial results and showed compelling outcomes for patients with a single tumor with a diameter <2 cm [25,27]. The results of this retrospective cohort study suggest a pretransplant patient selection based on radiologic tumor characteristics like the organ allocation rules in hepatocellular carcinoma (HCC) [28,29]. Indeed, a recent working group report from the International Liver Transplantation Society (ILTS) Transplant Oncology Consensus Conference stated that OLT might be considered in patients with cirrhosis and iCCA ≤2 cm but also in otherwise irresectable tumors in non-cirrhotic livers after neoadjuvant therapy with stable disease [30]. However, especially in the current era of increasing organ shortage, the debate on the role of OLT for iCCA needs to be supported by comparable high-quality outcome data after surgical resection from expert centers especially with respect to radiological tumor characteristics which still seems to be the most pragmatic way to identify ideal OLT candidates. 

Thus, we aimed to investigate the prognostic value of preoperative characteristics focusing on radiological features for long-term oncological outcome in iCCA patients.

## 2. Materials and Methods

### 2.1. Patients

Between 2010 and 2020, all consecutive patients with localized iCCA with no signs of systemic disease who were treated with liver resection at the University Hospital RWTH Aachen (UH-RWTH), were included in this study. The study was conducted in accordance with the requirements of the Institutional Review Board of the RWTH Aachen University (EK 169/21), the current version of the Declaration of Helsinki, and the good clinical practice guidelines (ICH-GCP). All clinical data were prospectively collected within an institutional hepatobiliary database.

### 2.2. Staging and Surgical Technique

All patients who were referred for surgical treatment of iCCA to our institution underwent a detailed clinical work up as previously described [7,31,32]. Particularly, tumor staging was assessed by multiphase computed tomography (CT) and/or magnetic resonance imaging (MRI). In patients with insufficient FLR scheduled for right-sided hepatectomy, right portal vein embolization (PVE) was carried out 2 to 4 weeks prior to surgery. The decision for surgery as primary treatment was made by an experienced hepatobiliary surgeon and approved by the local interdisciplinary tumor board. The surgical procedure was carried out as previously described [7,31,32,33]. In case of iCCA infiltrating the liver hilum, a “no-touch” hilar en bloc resection approach, as defined by liver resection with portal vein resection and/or arterial resection on demand, was performed. Peripherally located iCCA were resected in accordance with common clinical standards. Lymphadenectomy comprising the pericholedochal, the periportal, the common hepatic lymph nodes, the posterior pancreaticoduodenal, and the celiac lymph nodes was routinely performed. Parenchymal transection was carried out as described using the Cavitron Ultrasonic Surgical Aspirator (CUSA^®^, Integra LifeSciences^®^, Plainsboro, NJ, USA). All surgical specimens were evaluated by an experienced board-certified staff pathologist und classified according to the 8th edition of the Union for International Cancer Control (UICC). No patients underwent further direct treatment except for adjuvant therapy in some cases. 

### 2.3. Statistical Analysis

The primary endpoint of this study was overall survival (OS), which was defined from the date of resection to the date of tumor-specific death or the last contact if the patient was alive. The secondary endpoint was recurrence-free survival (RFS), which was defined as the period from surgery to the date of first recurrence. Patients without tumor recurrence were censored at the time of death or at the last follow-up. The associations of OS and RFS with clinico-pathological characteristics were assessed using univariate and multivariable Cox regression analyses in a forward selection model. Survival curves were generated by the Kaplan–Meier method and compared with the log rank test. Median follow-up was assessed with the reverse Kaplan–Meier method. The level of significance was set to *p* < 0.05 and *p*-values were given for two-sided testing. Analyses were performed using SPSS Statistics 24 (IBM Corp., Armonk, NY, USA).

## 3. Results

### 3.1. Patient Cohort

Between 2010 and 2020, a total of 139 patients with iCCA underwent curative-intent surgery at our hepatobiliary center. The study cohort comprised 61 male (43.9%) and 78 female (56.1%) individuals with a mean age of 65 years and a mean BMI of 26 kg/m^2^. The majority of patients were classified as ASA (American Society of Anesthesiologists classification) III or higher (57.2%, 79/138). Neoadjuvant therapy was applied in a small subset of patients (9.4%, 13/139). Major hepatectomies were necessary in 76.3% of the cases (106/139). R0 resection was achieved in 121 patients (88.4%). Major complications (≥Clavien–Dindo IIIa) after surgery were observed in 57/139 patients (41%). The pathological workup of the surgical specimen revealed nodal metastases in 36.7% (47/139) of the patients. 

Preoperative radiological characteristics were extensively assessed. The number of nodules was 1 in 70.5% (98/139), 2–3 in 12.2% (17/139), 4–5 in 7.2% (10/139), and >5 in 10.1% (14/139) of the cohort. Further, the largest tumor diameter was ≤3 cm in 12.9% (18/139), >3 cm and ≤5 cm in 15.8% (22/139), >5 cm and ≤1 0 cm in 42.4% (59/139), >10 cm and ≤15 cm in 21.6% (30/139), and >15 cm in 7.2% (10/139) of the patients. Macrovascular invasion was detected in 41% of the individuals (57/139). Based on the proposed radiologic risk stratification, 1 nodule ≤ 3 cm was observed in 12.9% (18/139) of all cases, while 1 nodule > 3 cm was present in 57.6% (80/139) and ≥2 nodules in 29.5% (41/139). 

More demographic and clinico-pathological details of the cohort are outlined in Table 1. Further, a comparative analysis with respect to the proposed radiologic risk stratification was carried out (Appendix A Table A1).

### 3.2. Survival Analysis 

**Figure 1:**
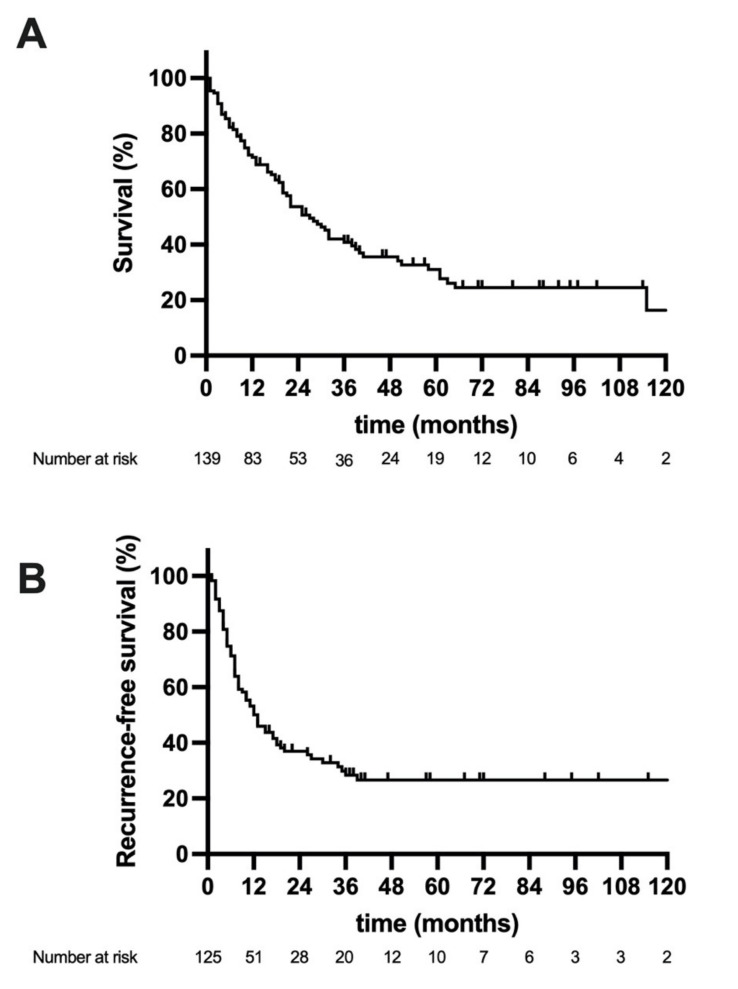
Oncological survival in intrahepatic cholangiocarcinoma the median overall survival (OS) (**A**) of the whole cohort was 25 months and the median recurrence-free survival (RFS) (**B**) 12 months.

**Figure 2:**
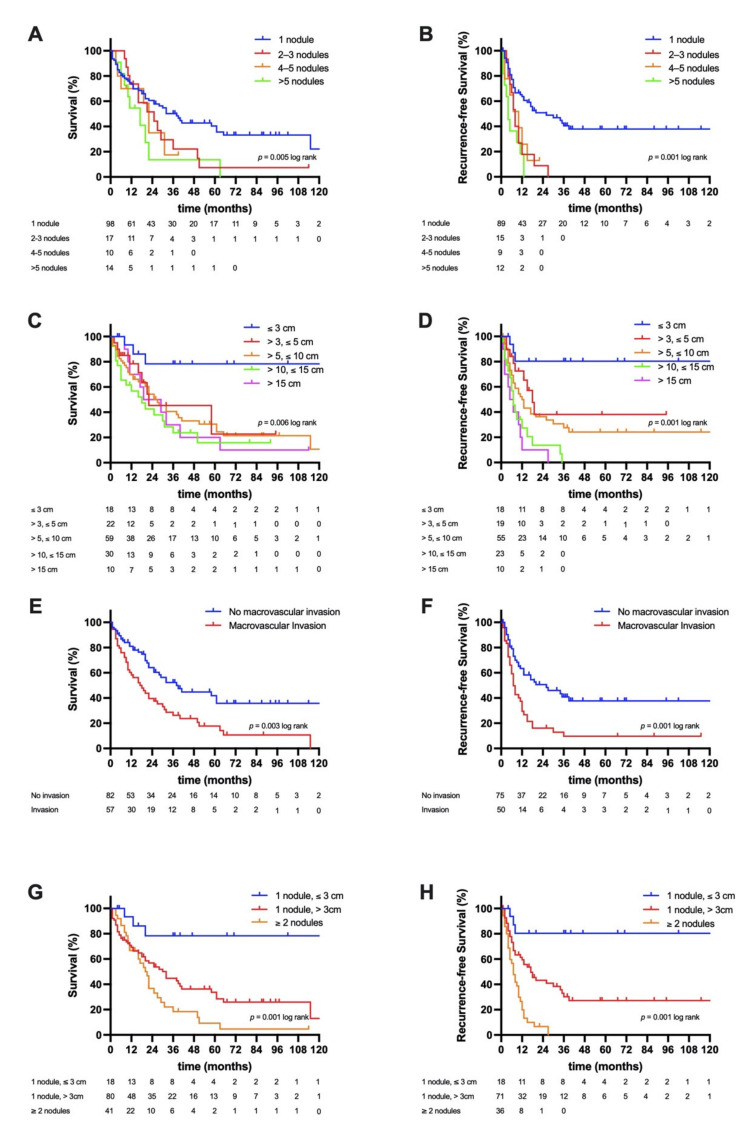
Oncological survival in intrahepatic cholangiocarcinoma stratified by radiologic features. (**A**) Overall survival (OS) stratified by number of nodules. The median OS was 32 months in patients with a single nodule, 21 months in patients with 2–3 nodules, 22 months in patients with 4–5, and 10 months in patients with >5 nodules. (**B**) Recurrence-free survival (RFS) stratified by number of nodules. The median RFS was 26 months in patients with a single nodule, 8 months in patients with 2–3 nodules, 10 months in patients with 4–5, and 4 months in patients with >5 nodules. (**C**) Overall survival stratified by largest tumor diameter. The mean OS was 142 months in patients with a largest tumor diameter ≤3 cm, while the median OS was 22 months in patients with >3 cm and ≤5 cm, 25 months in patients with >5 cm and ≤10 cm, 12 months in patients with >10 cm and ≤15 cm, and 19 months patients with >15 cm. (**D**) Recurrence-free survival stratified by largest tumor diameter. The mean RFS was 144 months in patients with a largest tumor diameter ≤3 cm, while the median RFS was 18 months in patients with >3 cm and ≤5 cm, 12 months in patients with >5 cm and ≤10 cm, 7 months in patients with >10 cm and ≤15 cm, and 5 months patients with >15 cm. (**E**) Overall survival stratified by macrovascular invasion. The median OS was 32 months in patients without macrovascular invasion and 16 months in patients with macrovascular invasion. (**F**) Recurrence-free survival stratified by macrovascular invasion. The median RFS was 20 months in patients without macrovascular invasion and 7 months in patients with macrovascular invasion. (**G**) Overall survival with respect to risk stratification. The mean OS was 142 months in patients with a single nodule ≤3 cm, while the median OS was 28 months in patients with a single nodule >3 cm and 19 months in patients with ≥2 nodules. (**H**) Recurrence-free survival with respect to risk stratification. The mean RFS was 144 months in patients with a single nodule ≤3 cm, while the median RFS was 17 months in patients with a single nodule >3 cm, and 7 months in patients with ≥2 nodules.

The median follow-up of the study cohort was 54 months and the overall number of deaths at 1, 3, and 5 years were 43 (31.2%), 74 (53.2%), and 81 (58.2%), respectively. The median OS of the whole cohort was 25 months (95% confidence interval (CI): 17–33), (3-year OS = 41%, 5-year OS = 31%), and the median RFS 12 months (95% CI: 8–16 (3-year RFS = 28%, 5-year CSS = 26%), Figure 1A,B). A Kaplan–Meier analysis with respect to radiologic risk stratification showed a mean OS of 142 months (95% CI: 107–178, 3-year OS = 78%, 5-year OS = 78%) in patients with a single nodule ≤3 cm, a median OS of 28 months (95% CI: 18–39, 3-year OS = 45%, 5-year OS = 33%) with a single nodule >3 cm, and a median OS of 19 months (95% CI: 10–28, 3-year OS = 18%, 5-year OS = 9%, *p* = 0.001 log rank) with ≥2 nodules (Figure 2G). Accordingly, the mean RFS was 144 months (95% CI: 110–179, 3-year RFS = 80%, 5-year OS = 80%) in patients with a single nodule ≤3 cm, a median RFS of 17 months (95% CI: 11–23, 3-year RFS = 30%, 5-year RFS = 27%) with a single nodule >3 cm, and a median RFS of 7 months (95% CI: 5–9, 3-year RFS = 0%, 5-year RFS = 0%, *p* = 0.001 log rank) with ≥2 nodules (Figure 2H). Further, a comparative analysis between patients surviving longer than the median OS and deceased patients was carried out (Appendix A Table A2)

Other radiologic criteria e.g., number of nodules (OS: *p* = 0.005 log rank, RFS: *p* = 0.001 log rank), largest tumor diameter (OS: *p* = 0.006 log rank, RFS = 0.001 log rank), and macrovascular invasion (OS: *p* = 0.003 log rank, RFS: *p* = 0.001 log rank) were further associated with survival in the cohort. A more detailed analysis for radiologic criteria is provided in Figure 2.

### 3.3. Cox Regression Analyses

In univariate analysis, number of nodules (*p* = 0.006), largest tumor diameter (*p* = 0.007), macrovascular invasion (*p* = 0.002), risk stratification (*p* = 0.001), gamma glutamyltransferase (GGT, *p* = 0.009), international normalized ratio (INR, *p* = 0.005), hemoglobin (*p* = 0.007), operative time (*p* = 0.028), intraoperative packed red blood cells (PRBC, *p* = 0.001), R1 resection (*p* = 0.022), microvascular invasion (MVI, *p* = 0.039), lympho-vascular invasion (LVI, *p* = 0.001), tumor grading (*p* = 0.001), pT category (*p* = 0.011), pN category (*p* = 0.001), intensive care unit (ICU) time (*p* = 0.001), duration of hospitalization (*p* = 0.001), and perioperative complications (*p* = 0.001) were associated with OS (Table 2). All variables showing *p* value < 0.1 were included in a multivariable Cox regression model (Table 3 and Table 4). Here, neoadjuvant therapy (HR = 7.26, *p* = 0.001), GGT (Exp(B) = 1.00, *p* = 0.007), LVI (HR = 5.65, *p* = 0.001), pT category (HR = 2.44, *p* = 0.004), and ICU time (Exp(B) = 1.04, *p* = 0.001) were identified as independent predictors of OS (Table 3). In a separate multivariable model based on preoperative characteristics, neoadjuvant therapy (HR = 3.46, *p* = 0.001), risk stratification (HR = 4.25 (1 nodule, >3 cm), HR = 5.97 (≥2 nodules), *p* = 0.011), GGT (Exp(B) = 1.00, *p* = 0.002), and INR (Exp(B) = 20.12, *p* = 0.014) were the independent predictors of OS (Table 4).

A similar approach was used to identify independent predictors of RFS. In univariate analysis, body mass index (BMI, *p* = 0.047), number of nodules (*p* = 0.001), largest tumor diameter (*p* = 0.001), macrovascular invasion (*p* = 0.001), risk stratification (*p* = 0.001), tumor localization (*p* = 0.003), bilirubin (*p* = 0.032), operative procedure (*p* = 0.008), intraoperative packed red blood cells (PRBC, *p* = 0.005), R1 resection (*p* = 0.025), MVI (*p* = 0.013), LVI (*p* = 0.004), pN category (*p* = 0.001), and perioperative complications (*p* = 0.024) were associated with RFS (Table 2). All variables showing *p* values < 0.1 were included in a multivariable Cox regression model (Table 3 and Table 4). Here, risk stratification (HR = 3.90 (1 nodule, >3 cm), HR = 13.16 (≥2 nodules), *p* = 0.001), and bilirubin (Exp(B) = 1.08, *p* = 0.007) were identified as independent predictors of RFS (Table 3). In a separate multivariable model based on preoperative characteristics, risk stratification (HR = 4.18 (1 nodule, >3 cm), HR = 11.07 (≥2 nodules), *p* = 0.001), and bilirubin (Exp(B) = 1.07, *p* = 0.015) were identified as independent predictors of RFS (Table 4).

## 4. Discussion

The surgical therapy for iCCA remains challenging due to the usually large tumor mass requiring major liver resection with the frequent need of “on demand” vascular reconstruction. Accordingly, the perioperative course of these patients is often burdened with notable perioperative morbidity and mortality [34,35]. Giving the steadily increasing age-standardized incidence of iCCA worldwide, offering curative-intent treatment is a major goal in therapy of this complex oncological disease [3]. As some iCCA patients might not be ideal candidates for liver resection due to technical irresectablility or an underlying liver disease, OLT has been recently discussed as a therapeutic alternative for selected cases with early iCCA based on radiological selection criteria including tumor size and number of nodules [25,27,36]. To facilitate the idea of patient selection based on preoperative available information, we assessed the prognostic ability of clinical and radiological characteristics for long-term outcome after curative-intent surgery for iCCA. 

In a large monocentric cohort of iCCA patients, we were able to demonstrate that preoperatively determined radiological characteristics e.g., number of nodules tumor diameter and macrovascular invasion can stratify oncological risk and are highly associated with RFS and OS. Particularly, we propose a risk stratification (1 nodule ≤3 cm, 1 nodule >3 cm and ≥2 nodules) that shows significant association with RFS in multivariable models considering a larger set of prognostic variables as well as and preoperative variables only. In addition, our risk stratification showed a good association with OS in a multivariable model when considering preoperative variables exclusively (Table 3 and Table 4). This observation translates into a 5-year OS of 78% in patients with a single nodule ≤3 cm, a 5-year OS of 33% in patients with a single nodule >3 cm and 5-year OS of 9% in patients with ≥2 nodules. The latter indicates not only the very good ability of our model to stratify patients into various risk groups, but also the compelling long-term outcome after surgical resection in early iCCA. Further, it must be emphasized that the survival figures in our low-risk subgroup sustained after 5 years and estimated 10-year OS was also 78%, indicating that most of these long-term survivors can be considered as curatively and definitively treated from an oncological point of view. However, this interesting observation on long-term outcomes might also be explained by the low incidence of nodal metastases which were present in only two cases (11.1%). Interestingly, patients with small iCCA are also the sub-cohort in which OLT is proposed as a treatment option [25,27].

OLT as a therapy concept for iCCA has recently gained novel interest among hepatobiliary surgeons. Particularly, Sapisochin et al. reported a small cohort of 29 patients who underwent OLT for HCC and were incidentally found to have iCCA in the explanted livers [37]. Here the 5-year OS in the whole cohort was 45% and, more importantly, 73% in the subgroup of patients with early iCCA (defined as a single nodule ≤ 2 cm). These survival figures were later confirmed by a multicenter cohort displaying a 5-year OS of 65% in patients with early iCCA [27]. As these survival figures appear slightly inferior to our results for even larger iCCA (defined as a single nodule ≤ 3 cm), at the first glance, our data may challenge the role of OLT in these patients in times where allografts are scarce. Based on these controversial findings we may draw at least a partial analogy to the ongoing discussion about resection versus transplantation for HCC which is certainly influenced by the possibility of liver resection in patients with underlying parenchymal liver disease as well as the scarcity of donor grafts especially in western Europe [38]. One might argue that in cases of severe underlying liver disease, the options for surgical treatment are limited due to the risk of PHLF. However, more than ¾ of patients in our cohort with a single nodule ≤3 cm were also diagnosed with an (preoperatively suspected) underlying liver disease that was confirmed by final histopathology (14/18 patients (77.8%) with cirrhosis in 2 patients (14.3%), fibrosis in 7 patients (50.0%), and NAFLD in 5 patients (35.7%). Moreover, the implementation of minimal-invasive techniques in liver surgery and the utilization of dynamic liver function tests broadened the traditional disease spectrum which can be addressed by liver resection [39,40,41,42,43]. Considering the limited data about OLT in iCCA, scarcity of donor grafts and the excellent outcome after surgical resection especially in very early tumors, the ideal patient selection for the entity warrants further evaluation. Our results indicate compelling long-term outcomes for small iCCA after liver resection. However, in case of severe cirrhosis with a small nodule in unfavorable location resulting in the requirement of a major resection, OLT might be an alternative option. In this scenario upfront OLT seems to be more suitable as these patients might not be able to undergo neoadjuvant treatment nor can be treated with any volume-modulating technique (e.g., ALPPS) due to their parenchymal damage and the reduced regenerative capacity of the liver. A corresponding clinical trial (NCT02878473) is currently being conducted to gather more explanatory data and expected to be analyzed for the first results in 5 years.

In tumors larger than 2 cm that are irresectable, the use of neoadjuvant therapy and subsequent OLT upon responsive or at least stable disease has been proposed [30]. In a recent publication, Lunsford et al. presented a small case series (*n* = 6) of locally advanced iCCA who underwent OLT [44]. Patients were eligible for OLT after a prolonged period of disease stability following treatment with neoadjuvant chemotherapy. While the 5-year OS was 83%, it must be noted that 50% of the patients developed disease recurrence during follow-up [44]. This excellent long-term OS can be, however, partially explained by a biological selection process. Among the initial 21 patients of this study, 12 individuals were listed for OLT and only 6 finally underwent OLT [44]. While the approach of Lunsford et al. is certainly interesting, it has to be acknowledged that only one patient who finally underwent OLT was considered irresectable due to the underlying liver disease whereas all other patients were not scheduled for surgery due to bilobar disease (*n* = 4) or tumor proximity to hilar structures (*n* = 1). With regard to neoadjuvant therapy, no prospective study has investigated its efficacy in iCCA so far. Current practice is mainly based on retrospective analyses such as the study by Le Roy et al. who reported a secondary resection rate of 53% (39 of 74 patients) of initially irresectable iCCA undergoing neoadjuvant chemotherapy [45]. More recently, Yadar et al. showed significantly better median survival (40.3 vs. 32.8 months) upon neoadjuvant vs. adjuvant chemotherapy using a propensity score-matched analysis from 278 vs. 700 patients from the National Cancer Database [46]. In contrast, neoadjuvant treatment was associated with impaired outcome in our own cohort which can be explained by our current strategy regarding perioperative treatment in iCCA. So far, we have only applied neoadjuvant therapy in case of irresectable local tumor extent. In turn, patients who undergo resection after neoadjuvant treatment in our cohort are supposed to have more advanced tumor stages. Nonetheless, the obvious advantage of neoadjuvant chemotherapy in iCCA is the biological selection since it is at least uncertain whether patients who progress upon systemic treatment would have benefited from initial upfront resection. However, one might also argue that patients with strongly impaired liver function who are not amendable for liver resection are hardly candidates for intensified systemic chemotherapy. In summary, the strategy of neoadjuvant chemotherapy before OLT might be an option for highly selected patients who almost preserved liver function but clearly needs to be further evaluated. 

The issue of impaired liver function in iCCA still remains an important problem. As mentioned above, more than ¾ of patients with tumor ≤3 cm in our study had liver damage. Similar, a recent multicentric analysis of 99 iCCA patients by Li et al. showed fibrosis in 26%, cirrhosis in 2%, and significant steatosis in 7% of patients who underwent ALPPS procedure. Although significant morbidity and mortality were observed, this study demonstrates the possibility for major liver surgery even in selected patients with chronic liver disease [24]. Regarding survival, the study by Li et al. reported 3-year and 5-year OS of 38.8% and 22.0%, respectively. When comparing these results to the study by Lunsford et al., one has to keep in mind the high drop-out rate in the latter trial (21 patients initially screened, 6 underwent OLT). Notably, the ALPPS cohort displayed tumor multifocality in 40% of patients, positive lymph nodes in 37% of the cases, and did not undergo a strict biological selection process by neoadjuvant therapy [24]. In summary, this data shows that even extended liver resection is possible in properly selected patients with iCCA instead of chronic liver disease. 

OLT in locally advanced iCCA should be considered as an individual concept as advanced iCCA is per se associated with a dismal oncological prognosis. Here, a biological selection by neoadjuvant systemic/interventional treatment can potentially identify patients with unfavorable surgical tumor characteristics but favorable tumor biology, as was also shown for other malignancies [47]. However, considering the rising technically feasibility of liver resection and the small number of available allografts, the term irresectability must be discussed critically. Further, in the trial of Lunsford et al., otherwise discarded liver grafts from extended criteria donors were utilized for the treatment of the patients. However, the potentially increased incidence of short- and long-term graft-related complications associated with the use of such “super” marginal allografts should be kept in mind when selecting patients for this procedure.

Additionally, of particular interest is our observation regarding the prognostic role of LVI in iCCA. Our group has recently reported the predictive value LVI in a large monocentric cohort of pCCA patients [36]. LVI of tumor cells, as defined by tumor cells present within a definite endothelial-lined space surrounding invasive carcinoma, is considered the hallmark feature of early tumor cell dissemination and has been identified as an important prognostic factor in patients with breast, esophageal, and rectal cancer [36,48,49,50]. However, the prognostic role of in iCCA and pCCA remains controversial [51,52,53]. Fischer et al. found that LVI might have an adverse effect on survival in patients with iCCA, but it must be noted that in this study, the authors did not differentiate between LVI and perineural infiltration [52]. Of note, LVI and peritumoral lymph vessel density are associated with nodal metastases in iCCA, suggesting LVI-dependent tumor cell dissemination through the lymphatic system in CCA [36,54]. This close association between LVI and lymph node metastases, which is traditionally considered to be a strong predictor of inferior outcome in iCCA, might explain why LVI was even superior to nodal status in our multivariable model [55,56,57,58].

Our results are in line with previous reports regarding oncological risk factors for iCCA. Multifocality, tumor size, and the invasion of major vessels are known risk factors for adverse oncological survival [36,55,56,57,58,59,60]. A large multicentric analysis revealed a median OS of 42 months in patients with unifocal disease which seems significantly superior to our subgroup with a single nodule with a median OS of 32 months [60]. However, a comparison of clinical variables of this multicenter cohort to our cohort displays our patients to be oncologically more advanced with nodal metastases and macrovascular invasion in more than one third of the patients while the cohort of Buettner et al. is characterized by nodal metastases in 17% and macrovascular invasion in only 10% of the cases. Interestingly, tumor size <5 cm is often used to identify patients with a better prognosis in previous reports [55,56,57,58]. However, our aim here was rather the identification of a low-risk group in which highly compelling outcomes can be reached, rather than fully exploring oncological limits. 

Like other retrospective clinical outcome studies, our analysis has several limitations, which should be considered when interpreting our data. First, all patients of this study underwent surgery in a single center in accordance with the author’s individual approach to iCCA and all data were obtained in a retrospective fashion. Second, radiologic imaging was not standardized as it would be in the setting of a controlled clinical trial and both multiphase CT and MRI were used to assess radiologic characteristics depending on the availability. Third, our study cohort comprises only 139 patients. Although being one of the larger single-institution cohorts from western countries, this is a rather small number of patients for statistical analysis and subgroup analysis. Hence, our claims about the identified low-risk group of early iCCA patients are somewhat limited regarding the statistical validity. Moreover, our findings lack confirmation in an extern validation cohort including higher patient numbers and potentially different surgical strategies. Finally, our low-risk group contains tumors ≤3 cm which is in contrast to the definition of the low-risk group of OLT in iCCA by Sapisochin et al. (≤2 cm). Therefore, future multicentric datasets are needed to support and validate our results.

Notwithstanding the aforementioned limitations, we have identified preoperative radiologic characteristics (nodule count, tumor diameter, macrovascular invasion) to be highly associated with long-term outcome in iCCA patients. Further, we could demonstrate that patients with a single nodule ≤3 cm display compelling OS and RFS. This also supports the fact that these patients benefit in particular from curative-intent liver resection and might therefore be preferably treated with surgery and not OLT in the future.

## Figures and Tables

**Table 1:** Patient characteristics.

Demographics	iCCA (*n* = 139)
Gender, m/f (%)	61 (43.9)/78 (56.1)
Age (years)	65 ± 12
BMI (kg/m^2^)	26 ± 5
Portal vein embolization, n (%)	12 (8.6)
ASA, *n* (%)	
I	3 (2.2)
II	56 (40.6)
III	74 (53.6)
IV	5 (3.6)
V	0
Preoperative chemotherapy	13 (9.4)
Radiological Characteristics	
Number of nodules, *n* (%)	
1	98 (70.5)
2–3	17 (12.2)
4–5	10 (7.2)
>5	14 (10.1)
Largest tumor diameter (cm), *n* (%)	
≤3	18 (12.9)
>3, ≤5	22 (15.8)
>5, ≤10	59 (42.4)
>10, ≤15	30 (21.6)
>15	10 (7.2)
Macrovascular invasion, *n* (%)	57 (41.0)
Site of invasion, *n* (%)	
Portal vein	16 (28.1)
Hepatic artery	3 (5.3)
Hepatic vein	11 (19.3)
Vena cava	2 (3.5)
Multiple	25 (43.9)
Risk stratification, *n* (%)	
1 nodule, ≤3 cm	18 (12.9)
1 nodule, >3cm	80 (57.6)
≥2 nodules	41 (29.5)
Tumor localization, *n* (%)	
Peripherally located	66 (47.5)
Central mass	73 (52.5)
Clinical Chemistry	
AST (U/L)	55 ± 90
GGT (U/L)	254 ± 328
Total bilirubin (mg/dL)	1.1 ± 3.6
Hemoglobin (g/dL)	13.0 ± 1.7
Platelet count (/nL)	263 ± 88
INR	1.0 ± 0.1
Prothrombin time (%)	99 ± 14
Operative Data	
Operative time (minutes)	311 ± 116
Operative procedure, *n* (%)	
Atypical/Monosegmentectomy	19 (13.7)
Bisegmentectomy	14 (10.1)
Hemihepatectomy	49 (35.3)
Extended hepatectomy	26 (18.7)
Trisectionectomy	17 (12.2)
Mesohepatectomy	4 (2.9)
ALPPS	10 (7.2)
Intraoperative PRBC, *n* (%)	44 (31.7)
Pathological Examination	
R0 resection, *n* (%)	121 (88.4)
pT category, *n* (%)	
I	57 (41.0)
II	57 (41.0)
III	15 (10.8)
IV	10 (7.2)
pN category, *n* (%)	
N0	81 (63.3)
N1	47 (36.7)
Tumor grading, *n* (%)	
G1	0
G2	87 (69.6)
G3	35 (28.0)
G4	3 (2.4)
MVI, *n* (%)	45 (33.8)
LVI, *n* (%)	30 (23.3)
Postoperative Data	
Intensive care, days	4 ± 9
Hospitalization, days	18 ± 17
Postoperative complications, *n* (%)	
No complications	51 (36.7)
Clavien–Dindo I	5 (3.6)
Clavien–Dindo II	26 (18.7)
Clavien–Dindo IIIa	29 (20.9)
Clavien–Dindo IIIb	10 (7.2)
Clavien–Dindo IVa	6 (4.3)
Clavien–Dindo IVb	1 (0.7)
Clavien–Dindo V	11 (7.9)
Oncologic Data	
Adjuvant chemotherapy, *n* (%)	43 (32.6)
Recurrence, *n* (%)	77 (56.6)
Median RFS, months (95% CI)	12 (8–16)
Median OS, months (95% CI)	25 (17–33)

Data presented as mean and standard deviation if not noted otherwise. ALPPS: Associating liver partition and portal vein ligation for staged hepatectomy; ASA: American society of anesthesiologists classification; AST: aspartate aminotransferase; BMI: body mass index; CSS: cancer-specific survival; GGT: gamma glutamyltransferase; iCCA; intrahepatic cholangiocarcinoma; INR: international normalized ratio; LVI: lympho-vascular invasion; MVI: microvascular invasion; OS: overall survival; PRBC: packed red blood cells; RFS: disease free survival; UICC: Union for International Cancer Control.

**Table 2:** Univariable analysis of recurrence-free and overall survival in intrahepatic cholangiocarcinoma.

		Recurrence-Free Survival (RFS)			Overall Survival (OS)		
	n	Median RFS, m (95% CI)	Hazard Ratio/Exp(B) (95% CI)	*p* Value	Median OS, m (95% CI)	Hazard Ratio/Exp(B) (95% CI)	*p* Value
**Sex**				.950			.107
Male	61	12 (7–17)	1		18 (1224)	1	
Female	78	13 (620)	.99 (.62–1.56)		31 (2042)	.70 (.46–1.08)	
**Age, years**				.985			.460
≤65	68	12 (717)	.99 (.97–1.01)		30 (1941)	1.00 (.99–1.03)	
>65	71	13 (422)			22 (1628)		
**BMI, kg/m^2^**				**.047**			.977
≤25	65	10 (616)	.95 (.90–1.00)		25 (1436)	1.00 (.95–1.05)	
>25	73	15 (921)			33 (1430)		
**ASA**				.759			.123
I/II	59	13 (620)	1		28 (1838)	1	
III/IV	79	12 (816)	1.07 (.68–1.69)		21 (1230)	1.40 (.91–2.16)	
**Neoadjuvant therapy**				.054			.091
No	126	13 (818)	1		27 (2034)	1	
Yes	13	7 (68)	2.00 (.99–4.06)		10 (416)	1.83 (.91–3.68)	
**Number of nodules, *n* (%)**				**.001**			**.006**
1	98	26 (844)	1		32 (1945)	1	
2–3	17	8 (610)	2.81 (1.51–5.24)		21 (537)	1.68 (.93–3.05)	
45	10	10 (317)	2.52 (1.12–5.67)		22 (1826)	1.70 (.77–3.79)	
>5	14	4 (26)	4.60 (2.31–9.16)		10 (516)	2.83 (1.50–5.36)	
**Largest tumor diameter (cm), *n* (%)**				**.001**			**.007**
≤3	18	144 (110179) *	1		142 (107178) *	1	
>3, ≤5	22	18 (1224)	3.71 (1.00–13.77)		22 (1628)	4.19 (1.15–15.31)	
>5, ≤10	59	12 (816)	5.40 (1.66–17.59)		25 (1832)	4.70 (1.46-15.20)	
>10, ≤15	30	7 (59)	11.00 (3.22–37.63)		12 (026)	7.42 (2.22–24.78)	
>15	10	5 (010)	13.76 (3.73–50.74)		19 (038)	5.52 (1.49–20.43)	
**Macrovascular invasion, *n* (%)**				**.001**			**.002**
No	82	20 (238)	1		32 (1945)	1	
Yes	57	7 (59)	2.27 (1.44–3.58)		16 (824)	1.99 (1.30–3.05)	
**Risk stratification, *n* (%)**				**.001**			**.001**
1 nodule, ≤3 cm	18	144 (110,179) *	1		142 (107,178) *	1	
1 nodule, >3 cm	80	17 (1123)	4.82 (1.49–15.62)		28 (1839)	4.44 (1.38–14.25)	
≥2 nodules	41	7 (59)	12.32 (3.69–41.11)		19 (1028)	7.39 (2.25–24.24)	
**Tumor localization, *n* (%)**				.091			**.003**
Peripherally located		32 (1747)	1		26 (547)	1	
Central mass		22 (1331)	1.47 (0.94–2.22)		9 (612)	2.01 (1.26–3.21)	
**AST, U/L**				.228			.060
≤35	71	17 (1322)	1.00 (1.00–1.01)		22 (1232)	1.00 (1.00–1.00)	
>35	67	10 (713)			25 (2736)		
**GGT, U/L**				.573			**.009**
≤120	65	18 (1125)	1.00 (1.00–1.00)		29 (1840)	1.00 (1.00–1.00)	
>120	69	9 (513)			18 (1125)		
**Bilirubin, mg/dL**				**.032**			.444
≤0.5	75	13 (719)	1.06 (1.01–1.13)		27 (1836)	.97 (.91–1.04)	
>0.5	62	12 (717)			20 (1228)		
**Platelet count, 1/nL**				.648			.290
≤250	66	13 (917)	1.00 (1.00–1.00)		22 (1727)	1.00 (.97–1.00)	
>250	71	12 (519)			28 (1739)		
**INR**				.765			**.005**
≤1	78	17 (925)	1.54 (.09–26.4)		28 (1937)	23.3 (2.5–214.2)	
>1	58	11 (517)			19 (1226)		
**Hemoglobin, g/dL**				.183			**.007**
≤13	67	9 (414)	.90 (.78–1.05)		16 (824)	.83 (.73–.95)	
>13	70	17 (1222)			32 (2044)		
**Operative time, min**				.101			**.028**
≤300	70	13 (719)	1.00 (1.00–1.00)		25 (1634)	1.00 (1.00–1.00)	
>300	69	10 (516)			25 (1535)		
**Operative procedure**				**.008**			.735
Atypical/Monosegmentectomy	19	64 (4386) *	1		54 (3575) *	1	
Bisegmentectomy	14	103 (43,164) *	1.40 (.37–5.21)		13 (026)	1.41 (.51–3.92)	
Hemihepatectomy	49	15 (723)	2.57 (.98–6.73)		25 (1337)	1.68 (.77–3.67)	
Extended hepatectomy	26	11 (418)	3.32 (1.21–9.11)		22 (046)	1.76 (.76–4.09)	
Trisectionectomy	17	5 (011)	5.05 (1.78–14.31)		27 (1539)	2.07 (.85–4.99)	
Mesohepatectomy	4	7 (311)	3.75 (.89–15.88)		18 (037)	1.42 (.30–6.74)	
ALPPS	10	8 (511)	5.55 (1.84–16.79)		18 (037)	2.11 (.81–5.48)	
**Intraoperative PRBC**				**.005**			**.001**
No	95	15 (1020)	1		29 (2137)	1	
Yes	44	7 (68)	1.97 (1.23–3.15)		11 (022)	2.04 (1.33–3.14)	
**R1 resection**				**.025**			**.022**
No	122	13 (818)	1		27 (2034)	1	
Yes	14	7 (68)	2.17 (1.10–4.27)		6 (012)	2.11 (1.11–3.99)	
**MVI**				**.013**			**.039**
No	87	13 (422)	1		32 (1945)	1	
Yes	45	10 (614)	1.82 (1.14–2.90)		20 (1624)	1.58 (1.02–2.50)	
**LVI**				**.004**			**.001**
No	99	13 (719)	1		36 (3547)	1	
Yes	30	6 (39)	2.24 (1.29–3.90)		4 (17)	4.00 (2.47–6.46)	
**Tumor grading**				.107			**.001**
G1/G2	87	13 (521)	1		36 (2349)	1	
G3/G4	37	12 (618)	1.57 (.91–2.71)		11 (616)	2.62 (1.62–4.22)	
**pT category**				.223			**.011**
I/II	114	13 (818)	1		27 (1836)	1	
III/IV	25	7 (113)	1.45 (.80–2.64)		10 (218)	1.89 (1.16–3.11)	
**pN category**				**.001**			**.001**
N0	81	18 (531)	1		40 (2555)	1	
N1	47	7 (410)	2.26 (1.38–3.70)		8 (313)	3.45 (2.19–5.44)	
**ICU time, days**				.737			**.001**
≤1	92	13 (917)	1.01 (.95–1.07)		31 (2339)	1.05 (1.03–1.07)	
>1	46	8 (511)			10 (023)		
**Hospitalization, days**				.105			**.001**
≤13	71	17 (1024)	1.01 (1.00–1.02)		32 (2341)	1.02 (1.01–1.03)	
>13	66	9 (612)			16 (725)		
**Perioperative complications**				**.024**			**.001**
Clavien–Dindo I/II	82	17 (1024)	1		36 (2646)	1	
Clavien–Dindo III/IV/V	57	8 (610)	1.70 (1.07–2.71)		10 (416)	2.43 (1.58–3.73)	
**Adjuvant therapy**				.693			.225
No	89	12 (519)	1		21 (1230)	1	
Yes	43	12 (816)	1.10 (.69–1.76)		28 (2333)	0.74 (.45–1.21)	

Various parameters are associated with overall or recurrence-free survival. * mean. ALPPS: associating liver partition and portal vein ligation for staged hepatectomy; ASA: American society of anesthesiologists classification; AST: aspartate aminotransferase; BMI: body mass index; GGT: gamma glutamyltransferase; iCCA; intrahepatic cholangiocarcinoma; ICU: intensive care unit; INR: international normalized ratio; LVI: lympho-vascular invasion; MVI: microvascular invasion; PRBC: packed red blood cells; OS: overall survival; RFS: recurrence-free survival. Significant changes are marked in bold.

**Table 3:** Multivariable analysis of recurrence-free and overall survival in intrahepatic cholangiocarcinoma.

	Recurrence-Free Survival (RFS)		Overall Survival (OS)	
	Hazard Ratio/Exp(B) (95% CI)	*p* Value	Hazard Ratio/Exp(B) (95% CI)	*p* Value
**Neoadjuvant therapy**				**.001**
No			1	
Yes			7.26 (2.58–20.46)	
**Risk stratification, n (%)**		**.001**		
1 nodule, ≤3 cm	1			
1 nodule, >3 cm	3.90 (.93–16.28)			
≥2 nodules	13.16 (3.07–56.39)			
**GGT, U/L**			1.00 (1.00–1.00)	**.007**
**Bilirubin, mg/dL**	1.08 (1.02–1.14)	**.007**		
**LVI**				**.001**
No			1	
Yes			5.65 (3.04–10.53)	
**pT category**				**.004**
I/II			1	
III/IV			2.44 (1.32–4.49)	
**ICU time, days**			1.04 (1.02–1.06)	**.001**

Various parameters are associated with overall (OS) or recurrence-free survival (RFS) in multivariable analysis. Only significant variables are shown. For OS, neoadjuvant therapy, number of nodules, largest tumor diameter, macrovascular invasion, risk stratification, tumor localization, AST, GGT, INR, operative time, intraoperative PRBC, R1 resection, MVI, LVI, tumor grading, pT category, pN category, intensive care unit (ICU) time, hospitalization, and perioperative complications were included in the multivariable model. For RFS, BMI, neoadjuvant therapy, number of nodules, largest tumor diameter, macrovascular invasion, risk stratification, tumor localization, bilirubin, operative procedure, intraoperative PRBC, R1 resection, MVI, LVI, tumor grading, pN category, and perioperative complications were included in the multivariable model. AST: aspartate aminotransferase; BMI: body mass index; GGT: gamma glutamyltransferase; INR: international normalized ratio; LVI: lympho-vascular invasion; MVI: microvascular invasion; PRBC: packed red blood cells; OS: overall survival. RFS: recurrence-free survival. Significant changes are marked in bold.

**Table 4:** Multivariable analysis of recurrence-free and overall survival in intrahepatic cholangiocarcinoma based on preoperative characteristics.

	Recurrence-Free Survival (RFS)		Overall Survival (OS)	
	Hazard Ratio/Exp(B) (95% CI)	*p* Value	Hazard Ratio/Exp(B) (95% CI)	*p* Value
**Neoadjuvant therapy**				**.001**
No			1	
Yes			3.46 (1.64–7.29)	
**Risk stratification, *n* (%)**		**.001**		**.011**
1 nodule, ≤3 cm	1		1	
1 nodule, >3 cm	4.18 (1.29–13.57)		4.25 (1.32–13.75)	
≥2 nodules	11.07 (3.32–36.91)		5.97 (1.80–19.78)	
**GGT, U/L**			1.00 (1.00–1.00)	.002
**Bilirubin, mg/dL**	1.07 (1.01–1.14)	.015		
**INR**			20.12 (1.82–222.03)	014

Various parameters are associated with overall (OS) or recurrence-free survival (RFS) in multivariable analysis. Only significant variables are shown. For OS, age, neoadjuvant therapy, number of nodules, largest tumor diameter, macrovascular invasion, risk stratification, tumor localization, AST, GGT, and INR were included in the multivariable model. For RFS, BMI, neoadjuvant therapy, number of nodules, largest tumor diameter, macrovascular invasion, risk stratification, tumor localization, and bilirubin were included in the multivariable model. AST: aspartate aminotransferase; BMI: body mass index; GGT: gamma glutamyltransferase; INR: international normalized ratio; OS: overall survival; RFS: recurrence-free survival. Significant changes are marked in bold.

## Data Availability

The data presented in this study are available upon request from the corresponding author. The data are not publicly available due to privacy reasons.

## Appendix A

**Table A1:** Patient characteristics stratified by risk factor.

Demographics	Single Nodule, ≤3 cm (*n* = 18)	Single Nodule, >3 cm (*n* = 80)	≥2 Nodules (*n* = 41)	*p* Value
Gender, m/f (%)	8 (44.4)/10 (55.6)	36 (45)/44 (55)	17 (41.5)/24 (58.5)	.932
Age (years)	62 (57–75)	68 (59–75)	63 (55–74)	.417
BMI (kg/m^2^)	27 (23–32)	25 (23–30)	25 (22–29)	.255
Portal vein embolization, *n* (%)	1 (5.6)	6 (7.5)	5 (12.2)	.605
ASA, *n* (%)				.051
I	0	3 (3.8)	0	
II	10 (58.8)	33 (41.3)	13 (31.7)	
III	5 (29.4)	41 (51.3)	28 (68.3)	
IV	2 (11.8)	3 (3.8)	0	
V	0	0	0	
Preoperative Chemotherapy	1 (5.6)	9 (11.3)	3 (7.3)	.655
**Radiological Characteristics**				
Number of nodules, *n* (%)				**.001**
1	18 (100)	80 (100)	0	
2–3	0	0	17 (41.5)	
4–5	0	0	10 (24.3)	
>5	0	0	14 (34.1)	
Largest tumor diameter (cm), *n* (%)				**.001**
≤3	18 (100)	0	0	
>3, ≤5	0	21 (26.3)	1 (2.4)	
>5, ≤10	0	42 (52.5)	17 (41.5)	
>10, ≤15	0	15 (18.8)	15 (36.6)	
>15	0	2 (2.5)	8 (19.5)	
Macrovascular invasion, *n* (%)	1 (5.6)	30 (37.5)	26 (63.4)	**.001**
Risk stratification, *n* (%)				
1 nodule, ≤3 cm	18 (100)	0	0	
1 nodule, >3 cm	0	80 (100)	0	
≥2 nodules	0	0	41 (100)	
Tumor localization, *n* (%)				**.001**
Peripherally located	17 (94.4)	40 (50.0)	16 (39.0)	
Central mass	1 (5.6)	40 (50.0)	25 (61.0)	
**Clinical Chemistry**				
AST (U/L)	27 (24–42)	32 (26–46)	39 (30–55)	.068
GGT (U/L)	44 (24–265)	138 (67–371)	138 (88–270)	**.027**
Total bilirubin (mg/dL)	0.5 (0.4–0.6)	0.5 (0.3–0.8)	0.5 (0.4–0.6)	.959
Hemoglobin (g/dL)	13.4 (12.7–14.4)	13.1 (11.9–14.2)	12.7 (11.9–14.2)	.335
Platelet count (/nL)	225 (165–272)	254 (202–308)	280 (206–334)	.229
INR	1.00 (0.95–1.09)	0.98 (0.94–1.04)	1.03 (0.96–1.10)	.079
Prothrombin time (%)	102 (88–109)	101 (94–111)	95 (86–107)	.064
**Operative Data**				
Operative time (minutes)	233 (154–331)	307 (161–376)	285 (228–366)	.073
Operative procedure, *n* (%)				**.002**
Atypical/Monosegmentectomy	4 (22.2)	13 (16.3)	2 (4.9)	
Bisegmentectomy	6 (33.3)	4 (5.0)	4 (9.8)	
Hemihepatectomy	7 (38.9)	27 (33.8)	15 (36.6)	
Extended hepatectomy	1 (5.7)	21 (26.8)	4 (9.8)	
Trisectionectomy	0	9 (11.3)	8 (19.5)	
Mesohepatectomy	0	2 (2.5)	2 (4.9)	
ALPPS	0	4 (5.0)	6 (14.6)	
Intraoperative PRBC, *n* (%)	1 (5.6)	29 (36.3)	14 (34.1)	**.038**
**Pathological Examination**				
R0 resection, *n* (%)	17 (94.4)	72 (90.0)	33 (82.5)	.169
pT category, *n* (%)				**.001**
I	18 (100)	36 (45.0)	3 (7.3)	
II	0	30 (37.6)	27 (65.9)	
III	0	7 (8.8)	8 (19.5)	
IV	0	7 (8.8)	3 (7.3)	
pN category, *n* (%)				**.049**
N0	14 (87.5)	46 (63.9)	21 (52.5)	
N1	2 (12.5)	26 (36.1)	19 (47.5)	
Tumor grading, *n* (%)				.415
G1	0	0	0	
G2	12 (80)	53 (70.7)	22 (62.9)	
G3	3 (20)	19 (25.3)	13 (37.1)	
G4	0	3 (4.0)	0	
MVI, *n* (%)	1 (6.7)	26 (32.9)	18 (46.2)	**.033**
LVI, *n* (%)	0	18 (23.4)	12 (32.4)	**.043**
**Postoperative Data**				
Intensive care, days	1 (1–2)	1 (1–3)	1 (1–5)	.221
Hospitalization, days	7 (5–12)	14 (8–26)	12 (10–25)	**.002**
Postoperative complications, *n* (%)				.486
No complications	12 (66.7)	26 (32.5)	13 (31.7)	
Clavien–Dindo I	1 (5.7)	2 (2.5)	2 (4.9)	
Clavien–Dindo II	2 (11.1)	15 (18.8)	9 (22.)	
Clavien–Dindo IIIa	3 (16.7)	18 (22.5)	8 (19.5)	
Clavien–Dindo IIIb	0	7 (8.8)	3 (7.3)	
Clavien–Dindo IVa	0	4 (5.0)	2 (4.9)	
Clavien–Dindo IVb	0	0	1 (2.4)	
Clavien–Dindo V	0	8 (10.0)	3 (7.3)	
**Oncologic Data**				
Adjuvant chemotherapy, *n* (%)	6 (33.3)	22 (70.3)	15 (36.6)	.698
Recurrence, *n* (%)	3 (16.7)	42 (53.2)	32 (82.1)	**.001**
Median RFS, months (95% CI)	144 (110–179) *	17 (11–23)	7 (5–9)	**.001**
Median OS, months (95% CI)	142 (107–178) *	28 (18–39)	19 (10–28)	.001

Data presented as median and interquartile range if not noted otherwise. * mean. Categorical data were statistically analyzed using the chi-squared test, fisher’s exact test, or linear-by-linear association in accordance with scale and number of cases. Continuous variables were compared by the Kruskal–Wallis test. ALPPS: Associating liver partition and portal vein ligation for staged hepatectomy; ASA: American society of anesthesiologists classification; AST: aspartate aminotransferase; BMI: body mass index; CSS: cancer-specific survival; GGT: gamma glutamyltransferase; iCCA: intrahepatic cholangiocarcinoma; INR: international normalized ratio; LVI: lympho-vascular invasion; MVI, microvascular invasion; OS: overall survival; PRBC: packed red blood cells; RFS: disease free survival; UICC: Union for International Cancer Control. Significant changes are marked in bold.

**Table A2:** Patient characteristics stratified by survivors and deceased patients.

Demographics	Survivors (*n* = 29)	Deceased Patients (*n* = 86)	*p* Value
Gender, m/f (%)	10 (34.5)/19 (65.5)	40 (46.5)/46 (53.5)	.258
Age (years)	60 (55–71)	68 (57–74)	.133
BMI (kg/m^2^)	26 (23–30)	25 (22–29)	.551
Portal vein embolization, *n* (%)	1 (3.4)	10 (11.6)	.195
ASA, n (%)			**.020**
I	2 (7.1)	1 (1.2)	
II	12 (42.9)	34 (39.5)	
III	11 (39.3)	50 (58.1)	
IV	3 (10.7)	1 (1.2)	
V			
Preoperative Chemotherapy	1 (3.4)	9 (10.5)	.246
**Radiological Characteristics**			
Number of nodules, *n* (%)			**.014**
1	27 (93.1)	53 (61.6)	
2–3	1 (3.4)	14 (16.3)	
4–5	1 (3.4)	7 (8.1)	
>5	0	12 (14.0)	
Largest tumor diameter (cm), *n* (%)			**.003**
≤3	8 (27.6)	3 (3.5)	
>3, ≤5	4 (13.8)	10 (11.6)	
>5, ≤10	12 (41.4)	41 (47.7)	
>10, ≤15	4 (13.8)	23 (26.7)	
>15	1 (3.4)	9 (10.5)	
Macrovascular invasion, *n* (%)	7 (24.1)	46 (53.5)	**.006**
Risk stratification, *n* (%)			**.001**
1 nodule, ≤3 cm	8 (27.6)	3 (3.5)	
1 nodule, >3 cm	19 (65.5)	50 (58.1)	
≥2 nodules	2 (6.9)	33 (38.4)	
Tumor localization, *n* (%)			.147
Peripherally located	18 (62.1)	40 (46.5)	
Central mass	11 (37.9)	46 (53.5)	
**Clinical Chemistry**			
AST (U/L)	21 (16–48)	34 (28–55)	.166
GGT (U/L)	77 (27–219)	186 (82–375)	**.005**
Total bilirubin (mg/dL)	0.5 (0.4–0.7)	0.5 (0.3–0.8)	.847
Hemoglobin (g/dL)	13.9 (13.0–14.4)	12.9 (11.8–14.2)	**.011**
Platelet count (/nL)	253 (216–280)	254 (191–316)	.992
INR	0.98 (0.92–1.05)	1.00 (0.96–1.07)	.087
Prothrombin time (%)	103 (93–115)	100 (90–106)	.051
**Operative Data**			
Operative time (minutes)	285 (188–350)	298 (230–376)	.298
Operative procedure, *n* (%)			**.228**
Atypical/Monosegmentectomy	8 (27.6)	8 (9.3)	
Bisegmentectomy	3 (10.3)	7 (8.1)	
Hemihepatectomy	8 (27.6)	30 (34.9)	
Extended hepatectomy	6 (20.7)	17 (19.8)	
Trisectionectomy	2 (6.9)	13 (15.1)	
Mesohepatectomy	1 (3.4)	2 (2.3)	
ALPPS	1 (3.4)	9 (10.5)	
Intraoperative PRBC, *n* (%)	4 (13.9)	38 (44.2)	**.003**
**Pathological Examination**			
R0 resection, *n* (%)	27 (93.1)	74 (86.0)	.459
pT category, *n* (%)			**.024**
I	18 (62.1)	22 (25.6)	
II	8 (27.6)	43 (50.0)	
III	2 (6.9)	12 (14.0)	
IV	1 (3.4)	9 (10.5)	
pN category, *n* (%)			**.001**
N0	23 (88.5)	42 (51.9)	
N1	3 (11.5)	39 (48.1)	
Tumor grading, *n* (%)			.098
G1	0	0	
G2	22 (88.0)	48 (61.5)	
G3	3 (12.0)	27 (34.6)	
G4	0	3 (3.8)	
MVI, *n* (%)	5 (19.2)	37 (44.6)	.052
LVI, *n* (%)	1 (4.0)	28 (35.0)	**.002**
**Postoperative Data**			
Intensive care, days	1 (1–2)	1 (1–4)	**.026**
Hospitalization, days	9 (7–15)	16 (10–30)	**.001**
Postoperative complications, *n* (%)			.083
No complications	15 (51.7)	23 (26.7)	
Clavien–Dindo I	1 (3.4)	2 (2.3)	
Clavien–Dindo II	7 (24.1)	18 (20.9)	
Clavien–Dindo IIIa	5 (17.2)	19 (22.1)	
Clavien–Dindo IIIb	0	8 (9.3)	
Clavien–Dindo IVa	1 (3.4)	5 (5.8)	
Clavien–Dindo IVb	0	0	
Clavien–Dindo V	0	11 (12.8)	
**Oncologic Data**			
Adjuvant chemotherapy, *n* (%)	10 (34.5)	22 (25.9)	.373
Recurrence, *n* (%)	11 (37.9)	61 (72.6)	**.001**

Data presented as median and interquartile range if not noted otherwise. Categorical data were statistically analyzed using the chi-squared test, fisher’s exact test, or linear-by-linear association in accordance with scale and number of cases. Continuous variables were compared by the Mann–Whitney U-test. Patients were defined as survivors if they were still alive during follow-up and lived longer than 25 months (the median OS of the overall cohort was 25 months). Patients were defined as deceased patients if they deceased during follow-up. ALPPS: associating liver partition and portal vein ligation for staged hepatectomy; ASA: American society of anesthesiologists classification; AST: aspartate aminotransferase; BMI: body mass index; CSS: cancer-specific survival; GGT: gamma glutamyltransferase; iCCA: intrahepatic cholangiocarcinoma; INR: international normalized ratio; LVI: lympho-vascular invasion; MVI: microvascular invasion; OS: overall survival; PRBC: packed red blood cells; RFS: disease free survival; UICC: Union for International Cancer Control. Significant changes are marked in bold.
